# Text Message Reminders Increase Appointment Adherence in a Pediatric Clinic: A Randomized Controlled Trial

**DOI:** 10.1155/2016/8487378

**Published:** 2016-12-29

**Authors:** Chia-Lei Lin, Nila Mistry, Jordana Boneh, Hong Li, Rina Lazebnik

**Affiliations:** ^1^Rainbow Babies and Children's Hospital, University Hospitals Cleveland Medical Center, Cleveland, OH, USA; ^2^Case Western Reserve University, Cleveland, OH, USA

## Abstract

*Background*. High no-show rates can burden clinic productivity and affect patient care. Although multiple studies have shown that text messages improve appointment adherence, very little research has focused on low-income and predominantly African American populations in resident clinic settings.* Objectives*. To determine whether incorporating a text message reminder reduces the no-show rate at an urban, pediatric resident clinic.* Methods*. A randomized controlled trial was conducted at a tertiary level ambulatory pediatric practice between August 2014 and February 2015. Following a demographic survey, 170 patients were enrolled. Patients were randomized into control or intervention groups. All patients received the standard voice message appointment reminder, but the intervention group additionally received a text message reminder. The primary outcome was no-show rate.* Results*. 95.3% of the participants were African American, and the overall no-show rate was 30.8%. No-show rate was significantly lower in the intervention group (23.5%) than the control group (38.1%) representing a difference of 14.6% (*p* = 0.04). No demographic factors were found to alter the association between no-show rate and text message intervention.* Conclusions*. Text message reminders effectively improve show rates at a resident pediatric practice with high no-show rates, representing a promising approach to improving appointment adherence.

## 1. Introduction

Missed clinic appointments present a significant burden to the health care system and prevent optimal care for patients. Pediatric visits are particularly important for several reasons. Growth at this age is rapid, and frequent well-child visits in the beginning years are crucial to evaluate development. Pediatricians monitor height and weight gain and motor and speech development, and administer vaccinations at proper times per recommendations set forth by the American Academy of Pediatrics. For the practice, a scheduled appointment slot represents time and effort invested by the clinic and scheduling personnel to prepare for the visit. In addition, failure to attend an appointment represents a missed opportunity for other patients that may require a timely follow-up, disrupting optimal care.

With many advances in technology in the last few decades, the use of electronic reminders has become increasingly prevalent. Text messages are a convenient mode of communication that is widely used and can effectively reach a large population. The most recent data show that 83% of American adults own a cell phone [1]. Although many practices exist in urban settings where patients may have limited resources, cell phone ownership remains prevalent. In addition to the convenience of receiving text messages, there is the added benefit of a saved reminder that can be accessed again, a benefit not always available with phone call reminders.

A 2013 Cochrane Review showed low to moderate quality evidence that text message reminders improve appointment attendance rates [2]. A recent BMJ article published in 2016 also showed that text-based electronic notifications improved adherence [3]. However, the studies included in these reviews were heterogeneous, encompassing different interests. The scope of research on text message studies is broad and continues to expand. Studies have been done in various subspecialty areas spanning from dental clinics to endoscopy clinics, and the outcomes studied include appointment adherence [4], medication adherence [5], vaccination rates [6], and chronic disease care [7]. Many of the results have been positive. For example, text messages improved attendance in adolescents attending outpatient mental health treatment [4]. A systematic review also showed that text messages improved patients' medication adherence, especially in those with chronic diseases such as HIV, asthma, diabetes, and heart disease [5]. However, there are also studies where text messages did not demonstrate a positive impact [8, 9]. Pediatric data regarding text messages is still limited. However, there is an emerging body of literature focusing on issues pertaining to the adolescent population, such as follow-up for sexually transmitted illnesses [10, 11], vaccination adherence [12, 13], and contraception adherence [14]. Still, there are not many studies focusing on text messages for pediatric appointment adherence. To date, there are no published studies regarding whether text messages are advantageous in an inner-city resident clinic population. With high no-show rates and lack of continuity, resident clinics could substantially benefit from an intervention, such as a text messages, that would improve appointment adherence.

Our resident clinic services mostly low-income families that are predominantly African American. There are no previous studies reporting the relationship between text message reminders and no-show rate in a predominantly African American population. Preliminary data from our institution indicated that more than 95% of families at the clinic have cell phones, 75% of families use text messaging daily, and 85% have unlimited text messages, suggesting that a text messaging reminder system would be feasible for our patient population. The survey also showed that while 32% preferred only a phone call reminder and 20% preferred just a text message reminder, 45% preferred both a text message and a phone call reminder. Based on the lack of text message studies performed with our patient demographics and our preliminary findings, this randomized controlled trial was initiated to study the hypothesis that a text message reminder would improve appointment adherence at our primary care pediatric practice.

## 2. Methods

### 2.1. Experimental Design

#### 2.1.1. Study Type

A randomized controlled trial was designed to test the impact of text reminders on attendance adherence at a pediatric resident clinic servicing an inner-city population. Patients who scheduled an appointment were randomized to receive text or not receive text, in addition to the standard clinic phone message, at a 1 : 1 allocation ratio using a simple randomization method.

#### 2.1.2. Participants

This study was approved by the hospital's Institutional Review Board. Patients and guardians were enrolled from a single, urban, pediatric primary care clinic staffed by residents at an academic center which serves an inner-city population. Data was collected on a rolling basis over a period of seven months from August 2014 to February 2015. All patients scheduled in the resident clinic were considered eligible except for those without cell phones and those whose projected subsequent appointment fell outside the planned period of data collection. Hence, only those whose next appointment fell within the seven-month period were included in the study. Guardians were either approached in the waiting room or referred to the researchers by physicians. Patients over age 18 consented for themselves, while guardians consented for all patients under age 16. For patients between ages 16–18, guardians consented but patients gave assent and could choose to receive a personal text message appointment reminder in addition to the one sent to their guardian.

#### 2.1.3. Survey

Once enrolled, participants filled out a survey. Survey questions included patient's name, birthday, age, gender, and race, participant's relationship to the patient, if their cell phone receives text messages, cell phone number, purpose of the current visit, projected time of next visit, purpose of the next visit, and highest level of education of the participant completing the form. Survey was conducted as a multiple choice questionnaire to standardize answers. There were no exclusions for the visit type at the time of enrollment, and there were no exclusions for the next scheduled appointment visit type. Visit types included well visits or immunization visits, sick visits, follow-up visits, and others. Sick visit reasons were colds, fevers, vomiting, diarrhea, rashes, and others. Follow-up reasons included newborn weight checks, neonatal bilirubin checks, and birth contraception, such as Depo-Provera injections.

#### 2.1.4. Intervention

On a weekly basis, the clinic schedule was surveyed to determine the scheduled appointment date for the participants. Preliminary survey data demonstrated that parents prefer texts within 3 days of the appointment. Hence, all the participants randomized to the intervention group received a text within 3 days of the appointment. The text message was sent from a private number. The Institutional Review Board and Office of Research Compliance did not approve the use of computer automated texting programs due to HIPAA concerns. Therefore, each text message was manually sent by the researchers performing the study from a prepaid cell phone with a unique number used only for the purposes of this study. The text messages were standardized to include the name of the pediatric practice along with the date and time of the appointment. The patient's name and other identifying data were not included in the text message. Recipients were asked to not reply to the sender, but the number of the pediatric practice was provided for patients to reschedule if needed.

#### 2.1.5. Outcome

Charts were periodically reviewed to determine which patients had arrived for their scheduled appointments. Data was also collected on rescheduled or canceled appointments in both groups. Our primary end-point was whether patients were no-show for their appointments.

### 2.2. Statistical Methods

#### 2.2.1. Sample Size Determination

Sample size was estimated based on internal institutional data from the previous year's no-show rate, which was 30%. We aimed to determine whether the no-show rate could be reduced to 15% by adding text reminders. Assuming a no-show rate of 30% in the group without text, a sample size of 160 (80 in each group) would achieve 90% power to detect a difference of 15% based on the two-sided *Z*-test with pooled variance and 5% significance level. To account for no cell phone ownership, no text message capacity in cell phone, or declined participation, we aimed to recruit approximately 200 patients.

#### 2.2.2. Randomization

**Figure 1:**
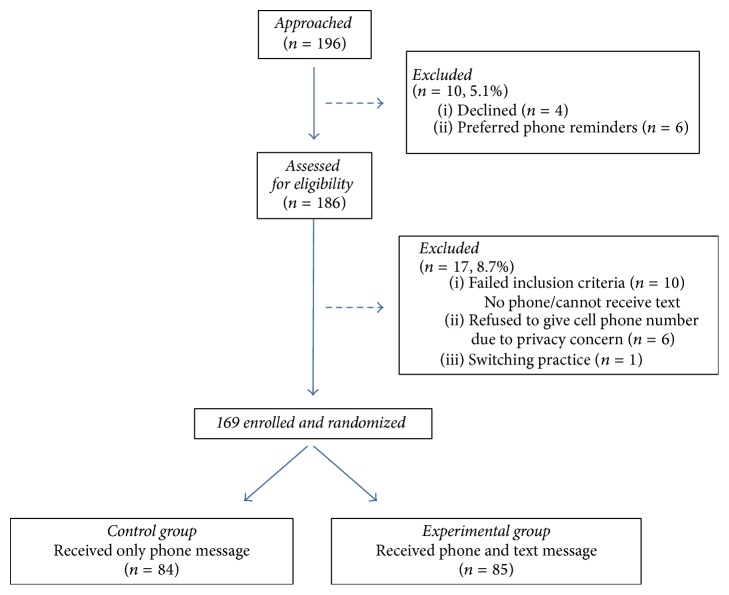
Flow chart of randomization.

Of the 196 parents or patients approached, 95% (186) agreed to participate (Figure 1). Those that declined to participate in the research study or stated their preference for only voice message reminders were immediately excluded. Of those assessed for eligibility, 17 (8.7%) were excluded. Ten participants did not meet inclusion criteria because they either did not own a cell phone or had a phone that could not receive text messages. One excluded patient planned to switch practices. Six excluded participants refused to provide their cell phone numbers for research purposes due to concerns for privacy. After exclusion, 169 were eligible, enrolled, and randomized to either the control or intervention group using simple randomization method. Patients were randomized on a rolling basis. The control group (*n* = 84) did not receive a text message reminder and only received the standard voice message reminder. The experimental group (*n* = 85) received a text message reminder in addition to the standard voice message reminder no more than three days prior to the appointment.

#### 2.2.3. Statistical Analysis

Caregiver's demographics and child's age between the two randomized groups were first compared using Chi-squared test, *t*-test, and Wilcoxon rank test to ensure the randomization was properly performed. Patients who did not show up for their appointment and did not cancel or reschedule the appointment were identified as “no-show.” Overall no-show rate was determined, and the rate between text sent and no text sent was compared using Chi-squared test. Child's age, reason for the visit, and caregiver's demographics were also compared between the two groups using Wilcoxon two-sample test or Chi-squared test. Child's age was dichotomized to less than 7 months of age and greater than or equal to 7 months of age, based on the age of frequent visits for well-child checks and immunizations. A multivariate logistic regression analysis was performed to estimate adjusted OR (95% CI) of kept/canceled/rescheduled visit by controlling for child's age, caregiver's education, and whether caregiver who received the text was mother or another guardian. All analyses were performed using SAS version 9.4. Two-sided *p* values are presented, and a *p* value less than 0.05 is considered statistically significant.

## 3. Results

To validate the randomization, caregiver's and child's characteristics of the control and intervention groups were examined. At baseline, there were no significant differences between the demographics of the control and intervention groups, including sex and race of the patient, median age of the patient, caregiver providing consent, and education level of the caregiver (data not shown).

**Figure 2:**
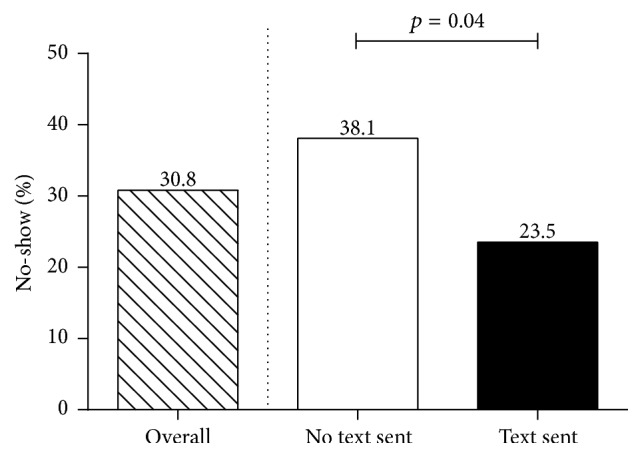
No-show rate for text sent and not sent.

The overall no-show rate during the period of data collection was 30.8%. The no-show rate was significantly lower in those who received text reminders with the standard phone message compared to those who only received phone message reminders (23.5% versus 38.1%, *p* = 0.04) (Figure 2).

In addition to text message intervention, associations between other factors and no-show were also determined (Table 1). One set of parameters examined was regarding the caregiver, including who consented to the study and their education level. In each of these cases, there was no significant association with the no-show population. Another set of parameters examined was related to the patient, including race, gender, age, and whether they were under 7 months old. Patient's age was of interest because children younger than 7 months old have more frequent visits. These factors were also not predictive of no-show events. The reason for the patient's visit at the time of enrollment and reason for the next appointment were not predictive of no-show events. On the other hand, text message intervention did in fact have a significant association in this univariate analysis.

Although not statistically significant, trends were noted when comparing the median patient age of the no-show group and other group (Table 1; 7.6 mo versus 5.3 mo, *p* = 0.29), the percentage of caregivers with some college education or higher (32.7% versus 45.3%, *p* = 0.12), and whether consent was obtained from the mother (90.4% versus 83.8%, *p* = 0.25). Hence, a multivariate logistic regression analysis was performed to determine whether the impact of text message reminders would remain significant after controlling for these factors. Analysis showed that when adjusted for age, caregiver education, and caregiver who gave consent, the intervention group was two times more likely (OR 2.12, CI 1.06–4.21) to keep, cancel, or reschedule the appointment (Table 2). Therefore, text message reminders can serve as an effective means to decrease pediatric patient no-show rates among low-income populations.

## 4. Discussion

Our results indicate that sending text message reminders is an effective means to improve appointment adherence at a pediatric resident clinic in an urban setting. Based on our pilot survey, where 33.8% of parents reported to have forgotten their scheduled visit, the text messages could serve as a valuable prompt for keeping the scheduled appointment. This is consistent with previous literature demonstrating the utility of text message appointment reminders on different patient populations [3, 4].

Our study is unique in that it focuses on a pediatric, urban, low-income population comprised mostly of African Americans. A literature search for similar studies with low-income populations largely points to studies done in developing countries [15], while many urban-based studies focus on issues such as HIV [16] and diabetes [17]. Few studies have looked at the impact of text messages on appointment adherence in an urban setting. There are even fewer studies that focus on pediatric populations in this setting. One study examined whether text message reminders improved rates of second dose of influenza vaccination in an urban clinic servicing mostly Latinos aged 6 months through 8 years, and it showed that the low-income, urban, minority population responded positively to text messages [18]. Many of the pediatric studies focus on adolescents, including one study utilizing text messaging for Depo-Provera injection reminders for urban adolescents and young adults, which also showed a positive effect with a text message reminder [14]. Our population is unique due to the relatively young age. Our findings add to the body of literature supporting the use of text message for pediatric patients in an urban setting.

To our knowledge, there are no studies done examining text message reminders in an inner-city resident clinic setting, where residents provide direct patient care under the supervision of faculty doctors. Continuity of care is challenging to achieve at a resident clinic [19]. We show here that sending a text message reminder can help increase the show rate and likely will improve continuity at the clinic. Our study results contrast with a similar study done in a dental school pediatric dentistry clinic, where text messaging did not improve appointment adherence [20]. This shows that more research needs to be done in this unique setting.

No-show can be a major burden to health care [21]. Missed appointments can lead to missed opportunities for disease detection and, in pediatrics, delayed vaccinations or recognition of developmental delays. Delayed care can potentially worsen outcomes for patients and increase health care costs for the government. A missed appointment also represents a financial burden to the clinic. A study performed at the VA Medical Center estimated that the average cost of no-show per patient was $196 in 2008 [21]. Financially, providing a text message is a promising and worthwhile way to reduce no-show rate. It is low cost and can be easily performed now with many automated systems on the market. However, the clinic must work with their legal system to ensure that the messages are delivered in a HIPAA compliant manner.

This study was performed in a clinic that serves mostly Medicaid patients. The results of this study are applicable to similar populations but may not be generalized to other populations or clinics with different demographics. This study was also limited by the nature of a resident clinic. Given that schedules cannot be made far in advance, this study naturally selected for infants and toddlers, who require more frequent visits. Another limitation is the lack of confirmation of text message receipt. The study did not allow for subjects to reply to the text message. Phone numbers change frequently; hence it could not be ascertained how many participants in the experimental group received the text message sent to them. A study looking at communication with adolescents via text messages in the context of Depo-Provera injections showed that phone number changes, unpaid cell phone bills, and phone loss significantly contribute to adolescent text nonresponsiveness rate [22].

Even with the addition of a text message, 24% of our patients still did not attend their appointment. Future research will be focused on this population. Another questionnaire via phone call can be administered to this population. Questions would include whether the text message was received, whether transportation was an issue, and so forth. Given the lower socioeconomic status of the study population, further research can delve into how limited resources play a role in show rates, so that the clinic can target ways to better serve the patient population. Potential future research can also look at parental response to the text message reminder and if frequent text messages would lead to eventual desensitization towards this type of reminder.

## 5. Conclusion

Text message reminders are an effective way to improve rate of attendance at an urban pediatric resident clinic with high no-show rates. This promising and modern technology represents an underutilized resource in primary care that will improve appointment adherence and potentially enhance patient care.

## Figures and Tables

**Table 1:** Comparison of characteristics in outcome groups.

Factor	No-show (*n* = 52)	Other ^*∗*^ (*n* = 117)	*p* value
*Caregiver parameters *			
Consent from mother (%)	47 (90.4)	98 (83.8)	0.25
College education or higher (%)	17 (32.7)	53 (45.3)	0.12

*Child parameters*			
Race, African American (%)	49 (94.2)	112 (95.7)	0.67
Gender, male (%)	26 (50.0)	52 (44.4)	0.50
Age (median, IQR)	7.6 (1.6, 23.8)	5.3 (1.6, 12.0)	0.29
Patients < 7 months old (%)	26 (50.0)	65 (55.6)	0.50

*Reason for visit at study enrollment*			
Well visit (%)	26 (50)	61 (52.1)	0.80
Sick visit (%)	14 (26.9)	25 (21.4)	0.43
Follow-up visit (%)	12 (23.1)	31 (26.5)	0.64

*Reason for visit of interest*			
Well visit (%)	34 (65.4)	81 (69.2)	0.62
Follow-up visit (%)	18 (34.6)	34 (29.1)	0.47

*Intervention*			
Text message (%)	20 (38.5)	65 (55.6)	0.04

^*∗*^Show, cancelled, or rescheduled.

**Table 2:** Factors associated with reduced no-show probability.

	Unadjusted		Adjusted ^*∗*^	
	OR (95% CI)	*p* value	OR (95% CI)	*p* value
*Randomization*				
Text sent	2.00 (1.03, 3.9)	0.04	2.12 (1.06, 4.21)	0.03
No text sent	1		1	

*Child's age (months)*				
Less than 7	1.25 (0.65, 2.41)	0.5	1.31 (0.66, 2.60)	0.44
7 or more	1		1	

*Education*				
College or higher	1.71 (0.86, 3.38)	0.13	1.70 (0.84, 3.43)	0.14
Lower than college	1		1	

*Caregiver*				
Mother	1.82 (0.64, 5.18)	0.26	2.19 (0.73, 6.55)	0.16
Other	1		1	

^*∗*^Adjusted factors listed in the same table.
